# Apelin-13 Enhances Arcuate POMC Neuron Activity via Inhibiting M-Current

**DOI:** 10.1371/journal.pone.0119457

**Published:** 2015-03-17

**Authors:** Dong Kun Lee, Jae Hoon Jeong, Seunghoon Oh, Young-Hwan Jo

**Affiliations:** 1 Division of Endocrinology, Department of Medicine, Albert Einstein College of Medicine of Yeshiva University, 1300 Morris Park Avenue, Bronx, NY, 10461, United States of America; 2 Department of Molecular Pharmacology, Albert Einstein College of Medicine of Yeshiva University, 1300 Morris Park Avenue, Bronx, NY, 10461, United States of America; 3 Department of Physiology, College of Medicine, Dankook University, Cheonan City, 330–714, Republic of Korea; University of Texas Health Science Center, UNITED STATES

## Abstract

The hypothalamus is a key element of the neural circuits that control energy homeostasis. Specific neuronal populations within the hypothalamus are sensitive to a variety of homeostatic indicators such as circulating nutrient levels and hormones that signal circulating glucose and body fat content. Central injection of apelin secreted by adipose tissues regulates feeding and glucose homeostasis. However, the precise neuronal populations and cellular mechanisms involved in these physiological processes remain unclear. Here we examine the electrophysiological impact of apelin-13 on proopiomelanocortin (POMC) neuron activity. Approximately half of POMC neurons examined respond to apelin-13. Apelin-13 causes a dose-dependent depolarization. This effect is abolished by the apelin (APJ) receptor antagonist. POMC neurons from animals pre-treated with pertussis toxin still respond to apelin, whereas the Gβγ signaling inhibitor gallein blocks apelin-mediated depolarization. In addition, the effect of apelin is inhibited by the phospholipase C and protein kinase inhibitors. Furthermore, single-cell qPCR analysis shows that POMC neurons express the APJ receptor, PLC-β isoforms, and KCNQ subunits (2, 3 and 5) which contribute to M-type current. Apelin-13 inhibits M-current that is blocked by the KCNQ channel inhibitor. Therefore, our present data indicate that apelin activates APJ receptors, and the resultant dissociation of the Gαq heterotrimer triggers a Gβγ-dependent activation of PLC-β signaling that inhibits M-current.

## Introduction

Apelin is a peptide, originally isolated from bovine stomach extracts and binds to the orphan G-protein-coupled apelin (APJ) receptor [1]. Apelin is considered one of adipokines as it is synthesized and secreted by adipocytes. The expression of apelin in fat cells is strongly regulated by the nutritional status in rodents [2]. Interestingly, recent studies have demonstrated that apelin-expressing cells are also present in the brain, in particular the hypothalamus [3–5]. Apelin-positive cells and its cognate APJ receptors are found in the paraventricular nucleus (PVN), dorsomedial nucleus (DMH), ventromedial nucleus (VMH) and arcuate nucleus (ARC) [3–5]. These hypothalamic regions are involved in the control of feeding behavior and glucose homeostasis [6–7]. Hence, the previous studies suggest that apelin has the ability to regulate energy homeostasis through alterations in hypothalamic neuronal activity. Indeed, intracerebroventricular (i.c.v.) administration of apelin induces the expression of c-fos, a marker of neuronal activity in the hypothalamus and differentially regulates glycemia depending on the nutritional state [8]. Moreover, acute i.c.v. injection of apelin decreases food intake [9–10].

There is abundant expression of apelin-positive cells in the ARC that is critical for sensing and integrating metabolic signals [11]. The ARC contains at least two types of neurons that oppositely regulate feeding behavior, such as anorexigenic proopiomelanocortin (POMC) / cocaine- and amphetamine-regulated transcript (CART) and orexigenic agouti-related peptide (AgRP) / neuropeptide Y (NPY)-expressing neurons. Interestingly, most apelin-positive neurons (~ 90%) in the ARC are POMC neurons, whereas only less than 10% of apelin-expressing neurons contain NPY [12]. Moreover, approximately half of POMC neurons express APJ receptor mRNAs and activation of APJ receptors induces the release of α-MSH from the hypothalamic explants in rodents [12]. Hence, the hypothalamic melanocortinergic system appears to be an important target that is regulated by apelin levels.

It has been demonstrated that the apelin/APJ receptor signaling pathway is mediated by both Gαi and Gαq proteins. For instance, activation of the APJ receptor inhibits adenylyl cyclase, lowering cAMP production [13] and stimulates phosphatidylinositol 3-kinases (PI3K) through pertussis toxin (PTX)—sensitive Gαi-mediated signaling [14]. In addition, the APJ receptor stimulates phospholipase C (PLC) and protein kinase C (PKC) via activating Gαq proteins [15]. In the hypothalamus, apelin increases nitric oxide (NO) release in fed mice [8] and induces a production of reactive oxygen species (ROS) [16]. Of particular interest is that POMC neuron activity is enhanced by increased PI3K signaling as well as ROS production [17–19]. It is thus plausible that apelin has the ability to increase POMC neuron excitability as like other adipokines such as leptin. In this study, we sought to determine the potential electrophysiological impact of apelin on POMC neurons in the ARC of the hypothalamus using whole-cell patch-clamp recordings.

## Materials and Methods

### Animals

All mouse care and experimental procedures were approved by the Institutional Animal Care Research Advisory Committee of the Albert Einstein College of Medicine. Mice used in these experiments were POMC-eGFP transgenic mice (The Jackson Laboratory).

### Slice preparation

Transverse brain slices were prepared from transgenic mice at postnatal age 28–35 days as described in the previous study [20]. Briefly, animals were anesthetized with isoflurane. After decapitation, the brain was transferred into a sucrose-based solution bubbled with 95% O_2_/5% CO_2_ and maintained at ~3°C. This solution contained the following (in mM): 248 sucrose, 2 KCl, 1 MgCl_2_, 1.25 KH_2_PO_4_, 26 NaHCO_3_, 1 sodium pyruvate, and 10 glucose. Transverse coronal brain slices (200 μm) were prepared using a vibratome. Slices were equilibrated with an oxygenated artificial cerebrospinal fluid (aCSF) for > 1 hr at 32°C before transfer to the recording chamber. The slices were continuously superfused with aCSF at a rate of 1.5 ml/min containing the following (in mM): 113 NaCl, 3 KCl, 1 NaH_2_PO_4_, 26 NaHCO_3_, 2.5 CaCl_2_, 1 MgCl_2_, and 5 glucose in 95% O_2_/5% CO_2_.

### Electrophysiological recordings

Brain slices were placed on the stage of an upright, infrared-differential interference contrast microscope (Olympus BX50WI) mounted on a Gibraltar X-Y table (Burleigh) and visualized with a 40X water-immersion objective by infrared microscopy (DAGE MTI camera). The POMC neurons were identified by the presence of enhanced green fluorescent protein (eGFP) resulting from the transgene. The internal solution contained the following (in mM): 115 K-Gluconate, 10 KCl, 5 CaCl_2_, 10 EGTA, 10 HEPES, 2 MgATP, 0.5 Na_2_GTP, and 5 phosphocreatine. All recordings were made at 30 ± 2°C. Membrane potentials were recorded in the presence of 6-Cyano-7-nitroquinoxaline-2,3-dione (CNQX, 10 μM), D-amino-phosphonovaleric acid (D-AP5, 50 μM) and picrotoxin (100 μM) with a Multiclamp 700B in whole cell configuration. Electrophysiological signals were low-pass filtered at 2–5 kHz, stored on a PC and analyzed offline with pClamp 10 software (Molecular devices, CA). For each recording, the firing rate and baseline membrane potential averaged from every 30 s were taken as one data point. 10 data points that represent stable activity levels were taken to calculate mean and standard deviation before and after application of apelin-13, respectively. A neuron was considered either depolarized or hyperpolarized if the change in membrane potential or firing rate induced by apelin-13 was ± 3 times the standard deviation prior to apelin-13 application. Both male and female animals were used in this study. In fact, the effect of apelin-13 on POMC neurons from males was similar to that observed in female mice.

The liquid junction potential between the cytosol and the pipette solution was calculated using the Henderson equation [21]. The calculated liquid junction potential was ~ +14 mV. The reversal potential (E_rev_) of the apelin-sensitive current was measured by ramping the membrane potential from -140 mV to -30 mV mV for 2 s in voltage-clamp configuration. In voltage clamp, we used two protocols to measure the deactivation and steady-state currents as described in the prior studies [22–23]. The deactivation protocol measured the current elicited during 500 ms voltage steps from -25 to -75 mV in 5 mV increments after a 300 ms prepulse to -20 mV from a holding potential of -60 mV in the presence of TTX (500 nM). The amplitude of M-current deactivation was measured as the difference between the initial and sustained current of the current trace. A series of command voltage steps from -80 to +10 mV in 10 mV increments for 1 s was applied from a holding potential of -90 mV and returning to -60 mV in the presence of TTX (500 nM) to examine the steady-state current.

Pertussis toxin (PTX, 0.5 μg) was injected directly into the midline arcuate nucleus of the hypothalamus (stereotaxic coordinates: AP, -1.2; ML, 0; DV, -5.5). In some experiments, we also co-injected Chicago Sky Blue to make sure that the injection site was correct. Based on the previous studies showing that 5-day treatment with PTX is sufficient to block Gαi signaling [24–26], animals were sacrificed 5 days post-injection. The mice injected with PTX were observed twice a day over a period of 5 days for clinical signs such as flaccid tail and hind limb weakness. No clinical signs were observed during 5-day treatment.

### Single-cell qPCR following single-cell whole-transcriptome amplification (WTA) of total RNA from individual POMC neurons

Single-cell samples were collected from brain slice preparations via aspiration into the patch pipette. The initial reverse transcription (RT) reaction was conducted after pressure ejection of the single cell samples into a microcentrifuge tube with REPLI-g WTA single cell kit (Qiagen). Samples were incubated in a total volume of 2.5 μl at 24°C for 5 minutes and cooled to 4°C. And then samples were incubated for 10 min at 42°C with 0.5 μl gDNA wipeout buffer prior to addition of 1.75 μl RT mix (RT mix: 0.25 μl oligo(dT) primer, 1 μl RT buffer, 0.25 μl random primer, 0.25 μl RT enzyme mix). The tubes were incubated at 42°C for 1 hr, and at 95°C for 3 min. The tubes then were incubated at 24°C for 30 min with 2.5 μl ligation mix (2 μl ligase buffer, 0.5 μl ligase Mix). The reaction was stopped by incubating at 95°C for 5 min. Samples were incubated at 30°C for 2 hrs after adding the amplification mix (7.25 μl buffer, 0.25 μl DNA polymerase) and at 65°C for 5 min.

Real-time PCR analysis was performed using a LightCycler 480 Instrument (Roche). qPCR reactions were prepared in a final volume of 20 μl containing 2 μl of reverse-transcribed cDNA, and 10 μl of SYBR Green master mix (Roche) in the presence of primers at 0.5 μM. A list of primer sets included: F5’-CCTGTTTGAGGATAGCAGCA-3’ and R5’-TAGCCTGCACAGGCAATATC-3’ for PLC β1, F5’-GCTCCTCGAAGCACATTCCT-3’ and R5’-AGCTTCCACCCGTTTTCGAT for PLC β2, F5’-GTGTGGAGCTGGATGTATGG-3’ and R5’-ACCTCAGTGGTCATGGTGAA-3’ for PLC β3, F5’-TGTGTTATGCGGTTCTTGGT-3’ and R5’-TGTCCAGCCGAGTACTGTTC-3’ for KCNQ2, F5’-GAGGAACAACGCCAAGTACA-3’ and R5’-AGAATCAAGCATCCCAGGAC-3’ for KCNQ3, F5’-ATCTCAAGAGGCCTGCAGTT-3’ and R5’-ATGGGTACCTGGGTAGCTTG-3’ for KCNQ5, and F5’-ACTTCTTCATTGCCCAAACC-3’ and R5’-GGCAAAGGTCACTACAAGCA-3’ for APJ. The target cDNAs were amplified for 50 cycles (94°C, 10 s; 60°C, 10 s; 72°C, 10 s) followed by 5 min at 72°C. After amplification, the PCR products were analyzed on 2% agarose gels.

### Statistics

Statistical analyses were performed on all POMC neurons examined. The mean values were reported from the neurons that respond to apelin-13 (GraphPAD 4.03 and Origin 8.5). Data were considered significantly different when the P value was < 0.05. All statistical results are given as mean ± S.E.M.

## Results

### Apelin-13 depolarizes POMC neurons in the ARC

**Fig 1:**
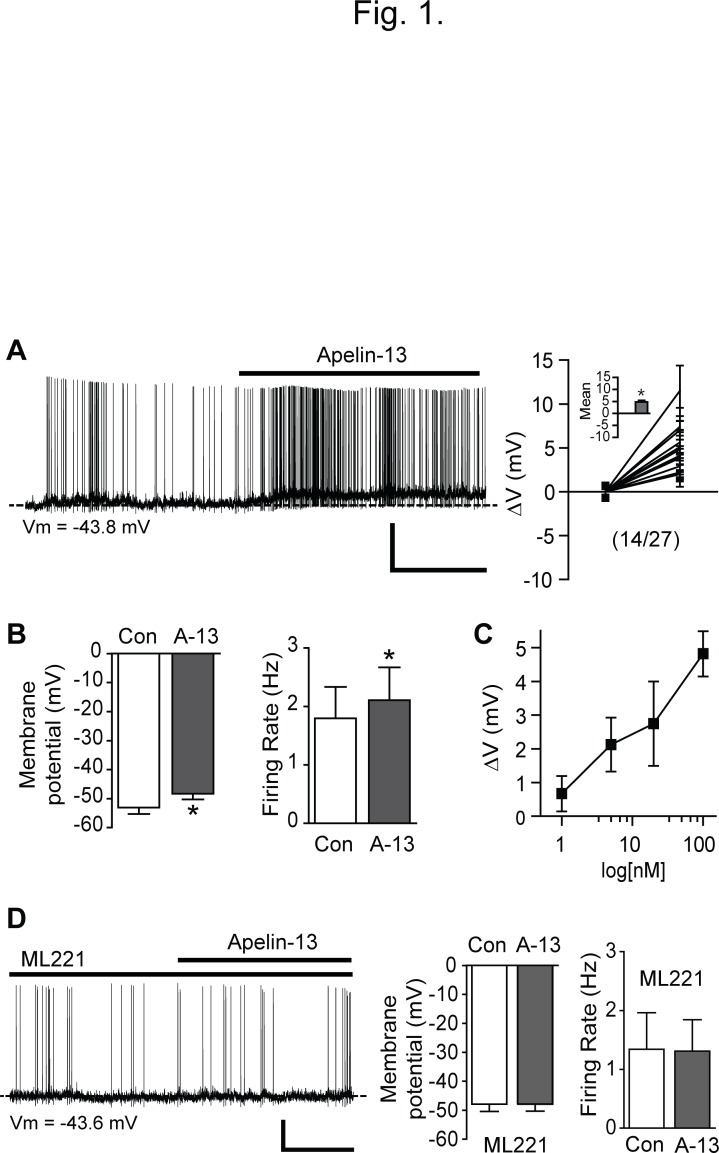
Apelin-13 depolarizes POMC neurons in the ARC. **(A)** Representative recording sample of the whole-cell membrane potential before, during and after treatment with apelin-13 after having blocked ionotropic glutamate and GABA receptors (left panel). Treatment with apelin-13 (100 nM) induced a depolarization and increased the firing rate of POMC neuron (right panel; mean change in membrane potential: +4.8 ± 0.7 mV, n = 14 neurons, p < 0.05). Scale bar: 20 mV, 2 min. **(B)** Pooled data of membrane potential and firing rate of POMC neurons with apelin-13 (A-13) (mean membrane potential: before -53.1 ± 2.2 mV vs. after -48.3 ± 1.9 mV, n = 14 neurons, p < 0.05; mean firing rate: before 1.8 ± 0.5 Hz vs. after 2.1 ± 0.6 Hz, n = 9 neurons, p < 0.05). **(C)** Apelin-13 causes a concentration-dependent depolarization of POMC cells. Depolarization by apelin-13 was determined at 1, 5, 20 and 100 nM in 6, 9, 6 and 14 neurons, respectively. **(D).** Representative whole-cell recording sample of the electrophysiological effect of apelin-13 in the presence of the APJ receptor antagonist ML221 (20 μM; left panel). Under these experimental conditions, POMC neurons did not respond to apelin-13 (middle and right panels; n = 11 neurons, p > 0.05). Scale bar: 20 mV, 2 min.

It has been demonstrated that half of POMC neurons in the ARC express the APJ receptor and that activation of the APJ receptor induces the release of α-MSH in *in vitro* preparations [12]. There are several biological active forms of apelin such as apelin -36, -17, and -13, which are derived from 77-amino acid precursor, preproapelin [27]. Among them, it has been shown that apelin-13 has greater biological activity than other forms [1]. Thus we used apelin-13 to study its role in the regulation of POMC neuron activity throughout our experiments. We included ionotropic glutamate and GABA receptor antagonists in aCSF to prevent presynaptic influences and determined whether apelin-13 alters the intrinsic electrical properties of POMC neurons. Under these experimental conditions, we found that approximately half of POMC neurons responded to apelin-13 (100 nM) with a depolarization. Fig. 1A represents a typical example of the response of POMC neurons to apelin-13. Treatment with apelin-13 (100 nM) significantly depolarized the membrane potential of POMC neurons examined (mean change in membrane potential: +4.8 ± 0.7 mV, n = 14 out of 27 neurons, p < 0.05; Fig 1A and B). In order to determine whether apelin-13 causes a concentration-dependent depolarization of POMC neurons, the magnitude of the effect was determined at 1, 5, 20 and 100 nM apelin-13. We found that the depolarizing effect was dose-dependent (n = 6, 9, 6 and 14 neurons, respectively; Fig. 1C). In addition to the depolarization, a small subset of POMC neurons were hyperpolarized by apelin-13 (mean change in membrane potential, -3.7 ± 1.3 mV; n = 3 out of 27 neurons examined).

We next investigated whether apelin-mediated depolarization is due to activation of the G-protein-coupled APJ receptor. The APJ receptor antagonist ML221 (20 μM) was added into aCSF. In the presence of ML221, apelin-13 (100 nM) had no effect on the membrane potential of POMC neurons (Fig. 1D; n = 11 neurons). There was no significant difference in membrane potential and firing rate before and after application of apelin-13 with ML221 (Vm: control, -47.9 ± 2.5 mV, apelin-13, -47.9 ± 2.5 mV, n = 11 neurons, p > 0.05; firing rate: control, 1.3 ± 0.6 Hz, apelin-13, 1.3 ± 0.5 Hz, n = 4 neurons, p > 0.05).

### PTX does not block the effect of apelin-13

**Fig 2:**
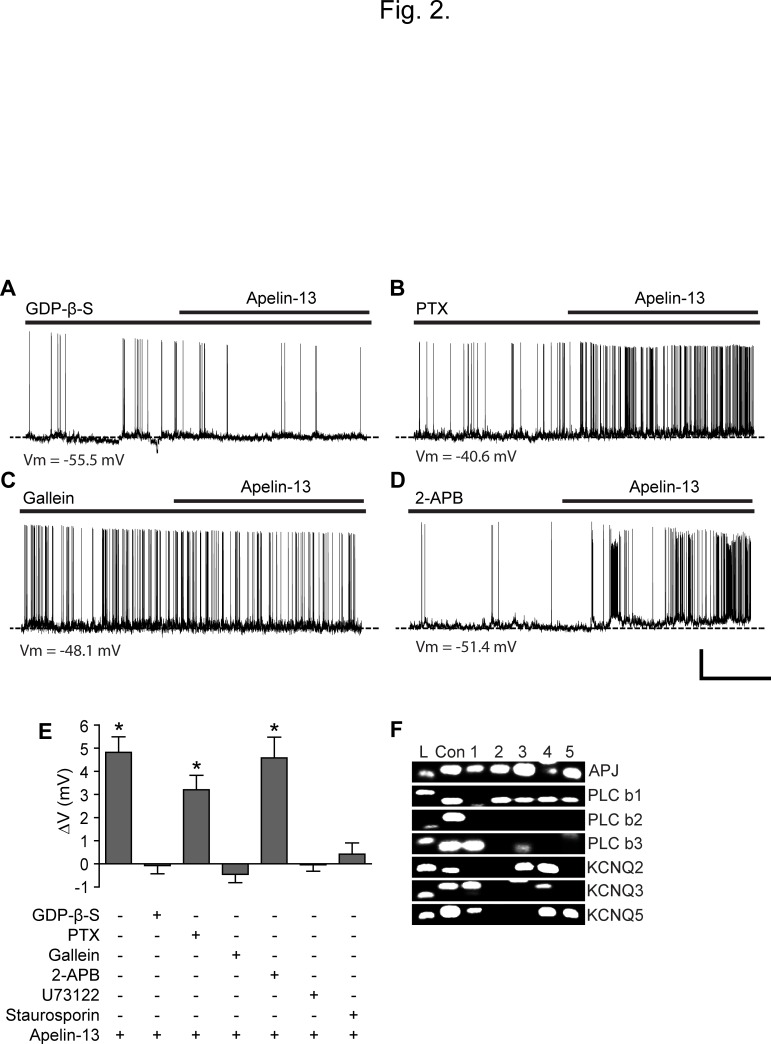
Apelin-13 excites POMC neurons via stimulating the Gβγ signaling pathway. **(A)** Representative recording sample of the whole-cell membrane potential of POMC neurons before, during, and after treatment with apelin-13 with GDP-β-S. GDP-β-S (2 mM) was directly introduced into the cell through patch pipette. Under these conditions, apelin-13 did not excite POMC neurons (n = 8 neurons). **(B-C)** Sample recording traces showing changes in membrane potential before, during, and after treatment with apelin-13 in animals pre-treated with PTX (B) and in the presence of gallein (C). Application of Apelin-13 (100 nM) effectively depolarized POMC neurons from animals pre-treated with PTX, whereas the same treatment did not change the membrane potential after having blocked Gβγ signaling with gallein. Scale bar: 20 mV, 2 min. **(D)** Representative recording sample showing that the TRPC channel antagonist 2-APB did not block the effect of apelin-13. Scale bar: 20 mV, 2 min. **(E)** Summary of the effects of apelin-13 in the presence of Gαi, Gβγ, PLC, PKC and TRPC inhibitors. GDP-β-S, Gallein, U73122, and staurosporin completely blocked the effect. POMC neurons remained to be sensitive to apelin-13 in the presence of PTX and 2-APB. **(F)** Single-cell qPCR analysis of individual POMC neurons. A representative gel illustrating the expression of the APJ receptor, PLC-β isoforms (1 and 3) and KCNQ subunits (2, 3 and 5) in POMC neurons (L: 100 bp ladder, con: positive control, 1–5: five different APJ receptor-expressing POMC neurons).

The APJ receptor is a G-protein-coupled receptor [28–29]. We intracellularly applied guanosine-5'-O-2-thiodiphosphate (GDP-β-S; 2 mM) into the cell as inclusion of a non-hydrolysable form of GDP through patch pipette prevents G protein-mediated signaling. Under such experimental conditions, POMC neurons did not responded to apelin-13 (Vm: control,- 52.2 ± 3.6 mV, apelin-13,- 52.3 ± 3.6 mV, n = 8 neurons, p > 0.05; Fig. 2A and E), consistent with the fact that this depolarization occurs dependently of G protein signaling.

We further examined whether apelin-13 stimulates Gαi or Gαq proteins in POMC neurons. PTX has been a very useful tool in determining the involvement of Gαi protein in signaling pathways [30]. We thus examined whether PTX abolishes apelin-induced depolarization of POMC neurons. To this end, we stereotaxically injected PTX into the ARC (see materials and methods) and then tested apelin-13 on POMC neurons 5 days post injection. We found that the membrane potential of POMC neurons from animals pre-treated with PTX was still depolarized by apelin-13. The mean change in membrane potential before and after apelin-13 was significantly different (+3.2 ± 0.6 mV, n = 6 out of 9 neurons, p < 0.05; Fig. 2B and E).

### Inhibition of PLC abolishes apelin-induced depolarization

As PTX did not block the depolarizing effect of apelin-13, the action of the APJ receptor would be mediated via Gαq coupling that stimulates PLC signaling. To examine the contribution of PLC, the PLC inhibitor was bath-applied. Under these experimental conditions, apelin-13 did not significantly shift the membrane potential of POMC neurons following treatment with the PLC inhibitor U73122 (10 μM; Fig. 2E). The mean membrane potential before and after application of apelin-13 was -51.1 ± 3.7 mV and -51.2 ± 3.8 mV, respectively (n = 6 neurons, p > 0.05). Stimulation of PLC signaling produces Inositol trisphosphate 3 (IP_3_) and diacylglycerol (DAG) which lead to an activation of protein kinase C (PKC) [31]. To examine the involvement of PKC, we directly introduced the protein kinase inhibitor staurosporine (100 nM) into POMC neurons via pipette solution. Under these experimental conditions, none of POMC neurons examined was depolarized by apelin-13 (Vm: control, -53.5 ± 3.9 mV, apelin-13, -53.1 ± 4.3 mV; n = 8 neurons, p > 0.05; Fig. 2E). Taken together, our data suggest that the APJ receptor in POMC neurons is preferentially coupled to Gαq, but not Gαi, proteins.

### Blockade of G-protein βγ subunit signaling abolishes the effect of apelin-13

The prior studies have demonstrated that not only the α-subunits of different G proteins but also the free Gβγ complex plays a role in signal transmission [32–34]. In particular, the βγ subunit can activate PLC independent of the G subunit. We intracellularly applied the Gβγ signaling blocker gallein (20 μM) into the cell to selectively restrain Gβγ signaling in POMC neurons. Under such experimental conditions, apelin-13 was no longer able to alter the membrane potential of POMC neurons (Vm: control, -49.8 ± 2.2 mV, apelin-13, -50.3 ± 2.2 mV; n = 7; p > 0.05; Fig. 2C and E), indicating that the observed depolarization occurs through direct activation of Gβγ signaling.

Interestingly, the βγ subunits appear to preferentially activate PLC-β1–3, but not PLC-γ isoforms [32–34]. Hence, we performed single-cell real time qPCR following single-cell whole transcriptome amplification of total RNA from individual POMC neurons. This method allowed us to determine whether individual POMC neurons co-express the APJ receptor and PLC-β1–3 isoforms. We found that 50% of POMC neurons examined expressed the APJ receptor (n = 5 out of 10 neurons; Fig. 2F). Our data further revealed that the majority of POMC neurons expressed PLC-β1 and-β3 isoforms (70%, 0% and 50% for PLC-β1,-β2 and-β3 isoforms, respectively; n = 7, 0 and 5 out of 10 neurons). More importantly, all APJ receptor-expressing POMC neurons also contained either PLC-β1 or PLC-β3 or both (Fig. 2F). These findings further suggest that the βγ subunits activate PLC-β isoforms, leading to a depolarization of POMC neurons.

### Apelin-13 inhibits M-current

It has been well documented that leptin as well as insulin stimulates canonical transient receptor potential (TRPC) channels through activation of the PLC signaling pathway, thereby depolarizing POMC neurons [17–18]. We thus investigated whether apelin depolarizes POMC neurons via activating TRPC channels. In the presence of the TRPC channel blocker 2-APB (100 μM) apelin remained effective in depolarizing POMC neurons (Fig. 2D and E). The mean membrane potential before and after application of apelin was significantly different (Vm: control, -47.8 ± 1.9 mV, apelin-13, -43.2 ± 2.0 mV; n = 5 neurons; p < 0.05). These data suggest that TRPC channels are not a downstream target of the apelin/APJ receptor signaling pathway.

**Fig 3:**
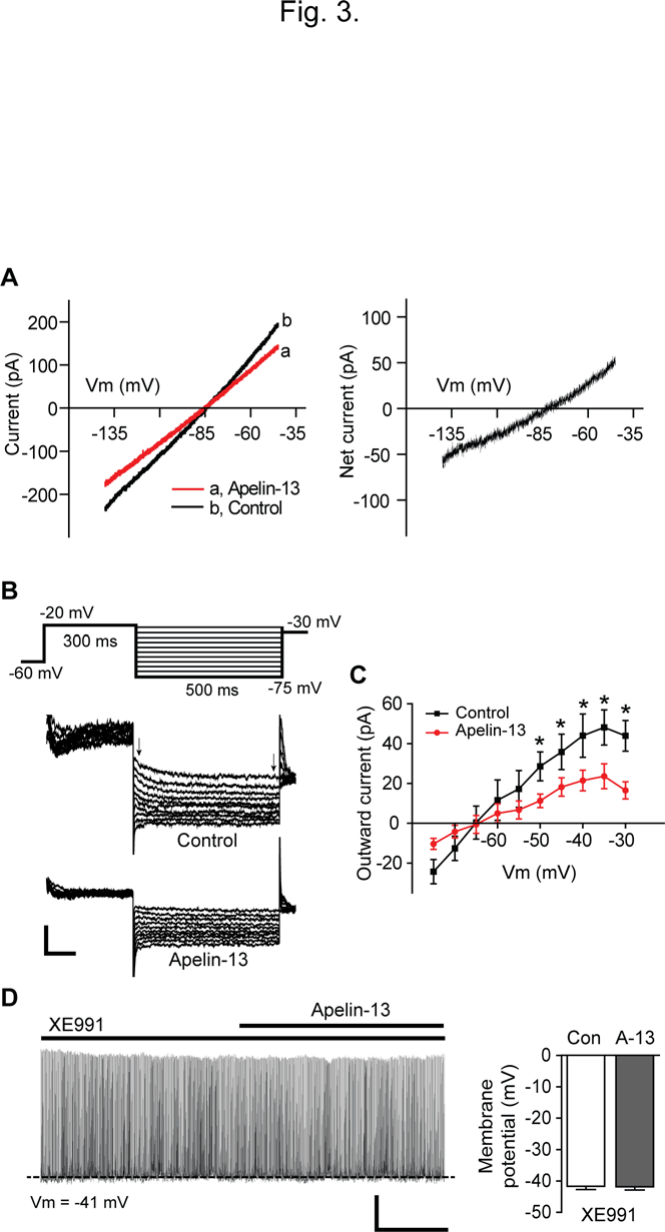
Apelin-13 inhibits M-type potassium current. **(A)** Current-voltage (I-V) relationship of the apelin-sensitive current. Left panel shows the whole-cell membrane currents in response to ramping the membrane potential from -140 mV to -30 mV before and after application of apelin-13 (100 nM). Right panel: The I-V relationship shows that the reversal potential of the apelin-sensitive current was -84.3 ± 1.4 mV (n = 10 neurons). **(B and C)** Sample membrane currents in response to voltage steps (a voltage jump to −20 mV was followed by steps from -25 mV to -75 mV; 5 mV step for 0.5 s) with and without apelin-13 at a holding potential of -60 mV (B). Apelin-13 inhibited the outward current (C). Scale bar: 100 pA, 100 ms. **(D)** Sample recording trace showing change in membrane potential before and during treatment with apelin-13 in the presence of the KCNQ channel blocker XE991 (left panel). Right panel: Pooled data of membrane potential of POMC neurons with apelin-13 (A-13) and XE991 (n = 12 neurons). Scale bar: 20 mV, 2 min.

Thus we further characterized the nature of the membrane depolarization. We measured the reversal potential (*E*_rev_) of the apelin-sensitive current by changing the membrane potential from −140 to −30 mV for 2 s in voltage-clamp configuration. The apelin-13-sensitive current was calculated by subtracting the current with apelin-13 from the control conditions. The *I*–*V* relationship revealed that the net current exhibited no rectification and that the *E*_*rev*_ of the apelin-sensitive current was −84.3 ± 1.4 mV (n = 10 neurons; Fig. 3A), a value close to the calculated K^+^ equilibrium potential under our experimental conditions. Our data suggest that apelin-13 inhibits potassium-mediated current.

Among potassium channels, KCNQ channels modulate neuronal excitability, action potential kinetics, and bursting firing [35–36]. Importantly these channels (KCNQ 2, 3, and 5 subunits) constitute M-type potassium current that is inhibited by activation of a number of G-protein-coupled receptors [35]. It has been shown that both neuropeptide Y (NPY) and POMC neurons in the ARC express KCNQ channels and that the regulation of M-current alters ARC neuronal excitability [22–23]. Indeed we also found that most APJ-expressing POMC neurons expressed KCNQ 2, 3 and 5 subunits, as shown in Fig. 2F.

We thus examined whether apelin-13 inhibits M-current in POMC neurons. To this end, we first measured the deactivation or relaxation of the whole-cell K^+^ currents induced by a well-established protocol in the presence of TTX (500 nM) [22–23]. The amplitude of M-current deactivation was measured as the difference between the initial and sustained current of the current trace (Fig. 3B). The outward K^+^ current evoked by this protocol was significantly decreased by apelin-13 (100 nM, n = 8 neurons). Treatment with apelin-13 significantly reduced the amplitude of the maximum K^+^ current that was recorded at—35 mV from 48.1 ± 8.8 pA to 23.6 ± 6.2 pA (n = 8 neurons, p < 0.05; Fig. 3B and C).

Next, we activated M-current with a series of command voltage steps from -80 to +10 mV in 10 mV increments for 1 s from a holding potential of -90 mV in the presence of TTX (500 nM) as described in the prior studies [22], [37]. Under these experimental conditions, the amplitude of the steady-state outward current at +10 mV was significantly decreased following treatment with apelin-13 (mean amplitude: control, 904 ± 109.5 pA; apelin-13, 700.4 ± 93.7 pA, n = 8 neurons, p < 0.05). The involvement of M-current was further examined by including the KCNQ channel inhibitor XE991 in aCSF. Treatment with XE991 (50 μM) significantly decreased the amplitude of the outward current at +10 mV (mean amplitude: control, 908.84 ± 183.76pA; XE991, 604.78 ± 118.84 pA, n = 5 neurons, p < 0.05), suggesting that POMC neurons express M-current. In the presence of XE991, treatment with apelin-13 no longer inhibited the outward current (mean amplitude: XE991, 604.78 ± 118.84 pA, XE991 + apelin-13, 592.51 ± 93.41 pA, n = 5 neurons, p>0.05).

Finally, we examined whether the KCNQ channel inhibitor abolishes the apelin-induced depolarization. In the presence of XE991 (50 μM), apelin-13 no longer changed the membrane potential of POMC neurons (Vm: XE991, -41.7 ± 1.1 mV, XE991 + apelin-13, -41.8 ± 1.0 mV; Fig. 3D), consistent with the fact that apelin-13 excites POMC neurons by inactivating KCNQ channels.

## Discussion

ARC POMC neurons are activated both directly and synaptically by circulating cues of energy surfeit such as glucose [38], insulin [17], and the fat-derived hormone leptin [39]. In this work, we outlined the cellular mechanisms underlying the depolarizing effect of apelin-13. We first showed that apelin-13 depolarized POMC neurons, which was completely abolished by the APJ receptor antagonist. Second, we demonstrated that this depolarization was due to a Gβγ-dependent activation of PLC-β signaling. Interestingly, all APJ receptor-positive POMC neurons expressed PLC-β isoforms (especially β1 and β3) that were a downstream target of Gβγ dimers. Third, activation of PLC-β signaling excited POMC neurons by inactivating XE991-sensitive M-type potassium channels. As described in recent studies of NPY and POMC neurons [22–23], regulation of the expression and activity of M-current in POMC neurons would contribute to the control of glucose homeostasis and food intake.

As apelin is synthesized and secreted from adipocytes, it is considered one of the adipokines [2]. Like other adipocyte-derived hormones such as leptin, apelin appears to be implicated in regulating glucose homeostasis and insulin sensitivity [8], [16], [40]. It has been proposed that the cellular basis of these metabolic effects involves a production of nitric oxide (NO) and reactive oxygen species (ROS) in the hypothalamus [8], [16]. However, changes in hypothalamic network activity would also contribute to these physiological processes. Indeed, central injection of apelin-13 induces the expression of c-fos in the ARC of fed animals [8], suggesting that apelin-13 is able to enhance the excitability of neurons in the ARC. POMC neurons in the ARC express the APJ receptor and activation of APJ receptors appears to induce the release of α-MSH [12]. It has been well documented that circulating nutrients, hormones, and neurotransmitters alter POMC neuron excitability by regulating a variety of channels such as K_ATP_ [38], [41], TRPC [17–18], [42], and KCNQ channels [23].

The APJ receptor has been shown to be coupled to Gαi and Gαq proteins. The activation of heterotrimeric G-proteins results in the exchange of GDP bound to the *α* subunit for GTP and the subsequent dissociation of the βγ subunit complex. And then effectors can be regulated by Gα only, by Gβγ only, by Gα or Gβγ or by Gα and Gβγ together [33]. In our preparations, apelin-13 did not lose the ability to alter POMC neuron activity in animals pre-treated with PTX, consistent with the fact that the observed depolarization does not require activation of Gαi proteins. Hence, it is possible that activation of the APJ receptor would trigger the Gαq signaling pathway. Alternatively, G protein βγ dimers would play a role in depolarizing POMC neurons independently of Gα proteins. Interestingly, apelin-13 no longer changed the membrane potential of POMC neurons after having blocked Gβγ signaling, consistent with the essential role of Gβγ subunits in the apelin/APJ receptor signaling pathway in POMC neurons.

The Gβγ subunit complex directly activates PLC-β, but not—γ, isoforms [32], [34], [43]. It seems likely that the majority of POMC neurons express PLC-γ isoforms [18]. Leptin and insulin stimulate the PLC-γ signaling pathway and the subsequent activation of PLC-γ isoforms leads to an opening of TRPC channels, thereby depolarizing POMC neurons [17–18]. Although the PLC inhibitor blocked the effect of apelin-13 in our preparations, the TRPC channel inhibitor did not influence apelin-induced depolarization of POMC neurons, suggesting the existence of other cellular mechanisms. In addition to the expression of PLC-γ isoforms, single-cell qPCR analysis of individual POMC neurons revealed that all APJ receptor-positive POMC neurons contained PLC-β isoforms, especially β1 and β3 isoforms. Our results thus suggest that PLC-β signaling appears to be a direct downstream target of the APJ receptor through the Gβγ subunit complex in POMC neurons.

The potassium channels underlying M-current are the KCNQ channel subunits (e.g. KCNQ2, KCNQ3, and KCNQ5) [35]. It has been shown that M-current controls the excitability of neurons in the ARC, including NPY and POMC neurons and is subject to modulation by nutrient availability and G protein-coupled receptors [22–23]. For instance, inhibition of M-current by serotonin increases POMC neuron excitability [23]. Importantly, M-type potassium channels are modulated by Gαq protein-coupled receptors [35–36] and is regulated specifically by PLC-β isoforms [36]. Moreover, PLC-β1–3 isoforms are regulated by Gα subunits of the Gq class as well as by Gβγ subunits [33]. In our preparations, the majority of POMC neurons having the APJ receptor and PLC-β isoforms expressed KCNQ subunits as well. Hence it looks likely that apelin-13 inhibits M-current through a Gβγ-dependent activation of PLC-β signaling.

We should emphasize that apelin-13 did not regulate TRPC channels in POMC neurons. It appears that, although both apelin and leptin increase POMC neuron excitability, they do not share common signaling pathways; one is PLC-γ signaling and the other is mediated by PLC-β isoforms. Therefore, it is plausible that each PLC isoform has a unique role in sensing and responding to diverse nutrients and hormones. This integration through PLC signaling would determine the physiological outcome of POMC neurons. In fact, isoform-specific deletion of signaling molecules, including PI3K and AMP-activated protein kinase (AMPK) alters responses of POMC and AgRP neurons to insulin and leptin [44–45]. As leptin and apelin receptors in POMC neurons improve glucose homeostasis and insulin sensitivity [8], [16], [40], [46], simultaneous activation of PLC-β and-γ signaling in POMC neurons could produce an additive effect on the magnitude of depolarization and ultimately strengthen the beneficial metabolic effects.

In summary, our results provide electrophysiological evidence that apelin-13 activates G-protein-coupled APJ receptors, and the resultant dissociation of the Gαq heterotrimer induces a Gβγ-dependent activation of PLC-β signaling, which in turn inhibits M current in POMC neurons. Our work suggests that the KCNQ channels in POMC neurons would be an alternative therapeutic target against obesity and type 2 diabetes as they are regulated by anorexigenic neurotransmitters and adipokines such as serotonin and apelin.

## References

[1] Tatemoto K, Hosoya M, Habata Y, Fujii R, Kakegawa T, Zou MX, et al Isolation and characterization of a novel endogenous peptide ligand for the human APJ receptor. Biochemical and biophysical research communications. 1998;251(2):471–6. 10.1006/bbrc.1998.9489 9792798

[2] Boucher J, Masri B, Daviaud D, Gesta S, Guigne C, Mazzucotelli A, et al Apelin, a newly identified adipokine up-regulated by insulin and obesity. Endocrinology. 2005;146(4):1764–71. Epub 2005/01/29. 10.1210/en.2004-1427 15677759

[3] Brailoiu GC, Dun SL, Yang J, Ohsawa M, Chang JK, Dun NJ. Apelin-immunoreactivity in the rat hypothalamus and pituitary. Neuroscience letters. 2002;327(3):193–7. Epub 2002/07/13. 12113910 10.1016/s0304-3940(02)00411-1

[4] Reaux A, Gallatz K, Palkovits M, Llorens-Cortes C. Distribution of apelin-synthesizing neurons in the adult rat brain. Neuroscience. 2002;113(3):653–62. 12150785 10.1016/s0306-4522(02)00192-6

[5] Pope GR, Roberts EM, Lolait SJ, O'Carroll AM. Central and peripheral apelin receptor distribution in the mouse: species differences with rat. Peptides. 2012;33(1):139–48. Epub 2011/12/27. 10.1016/j.peptides.2011.12.005 22197493 PMC3314948

[6] Sternson SM. Hypothalamic survival circuits: blueprints for purposive behaviors. Neuron. 2013;77(5):810–24. 10.1016/j.neuron.2013.02.018 23473313 PMC4306350

[7] Marino JS, Xu Y, Hill JW. Central insulin and leptin-mediated autonomic control of glucose homeostasis. Trends in endocrinology and metabolism: TEM. 2011;22(7):275–85. 10.1016/j.tem.2011.03.001 21489811 PMC5154334

[8] Duparc T, Colom A, Cani PD, Massaly N, Rastrelli S, Drougard A, et al Central apelin controls glucose homeostasis via a nitric oxide-dependent pathway in mice. Antioxidants & redox signaling. 2011;15(6):1477–96. 10.1089/ars.2010.3454 21395477

[9] Clarke KJ, Whitaker KW, Reyes TM. Diminished metabolic responses to centrally-administered apelin-13 in diet-induced obese rats fed a high-fat diet. Journal of neuroendocrinology. 2009;21(2):83–9. 10.1111/j.1365-2826.2008.01815.x 19076266

[10] Sunter D, Hewson AK, Dickson SL. Intracerebroventricular injection of apelin-13 reduces food intake in the rat. Neuroscience letters. 2003;353(1):1–4. Epub 2003/12/04. 14642423 10.1016/s0304-3940(03)00351-3

[11] van den Top M, Spanswick D. Integration of metabolic stimuli in the hypothalamic arcuate nucleus. Progress in brain research. 2006;153:141–54. 10.1016/S0079-6123(06)53008-0 16876573

[12] Reaux-Le Goazigo A, Bodineau L, De Mota N, Jeandel L, Chartrel N, Knauf C, et al Apelin and the proopiomelanocortin system: a new regulatory pathway of hypothalamic alpha-MSH release. American journal of physiology Endocrinology and metabolism. 2011;301(5):E955–66. 10.1152/ajpendo.00090.2011 21846903

[13] Reaux A, De Mota N, Skultetyova I, Lenkei Z, El Messari S, Gallatz K, et al Physiological role of a novel neuropeptide, apelin, and its receptor in the rat brain. Journal of neurochemistry. 2001;77(4):1085–96. 11359874 10.1046/j.1471-4159.2001.00320.x

[14] Masri B, Morin N, Cornu M, Knibiehler B, Audigier Y. Apelin (65–77) activates p70 S6 kinase and is mitogenic for umbilical endothelial cells. FASEB journal: official publication of the Federation of American Societies for Experimental Biology. 2004;18(15):1909–11. 10.1096/fj.04-1930fje 15385434

[15] Szokodi I, Tavi P, Foldes G, Voutilainen-Myllyla S, Ilves M, Tokola H, et al Apelin, the novel endogenous ligand of the orphan receptor APJ, regulates cardiac contractility. Circulation research. 2002;91(5):434–40. 12215493 10.1161/01.res.0000033522.37861.69

[16] Drougard A, Duparc T, Brenachot X, Carneiro L, Gouaze A, Fournel A, et al Hypothalamic apelin/reactive oxygen species signaling controls hepatic glucose metabolism in the onset of diabetes. Antioxidants & redox signaling. 2014;20(4):557–73. Epub 2013/07/25. 10.1089/ars.2013.5182 23879244 PMC3901354

[17] Qiu J, Zhang C, Borgquist A, Nestor CC, Smith AW, Bosch MA, et al Insulin excites anorexigenic proopiomelanocortin neurons via activation of canonical transient receptor potential channels. Cell metabolism. 2014;19(4):682–93. 10.1016/j.cmet.2014.03.004 24703699 PMC4183666

[18] Qiu J, Fang Y, Ronnekleiv OK, Kelly MJ. Leptin excites proopiomelanocortin neurons via activation of TRPC channels. The Journal of neuroscience: the official journal of the Society for Neuroscience. 2010;30(4):1560–5. Epub 2010/01/29. 10.1523/JNEUROSCI.4816-09.2010 20107083 PMC3095824

[19] Diano S, Liu ZW, Jeong JK, Dietrich MO, Ruan HB, Kim E, et al Peroxisome proliferation-associated control of reactive oxygen species sets melanocortin tone and feeding in diet-induced obesity. Nature medicine. 2011;17(9):1121–7. Epub 2011/08/30. 10.1038/nm.2421 21873987 PMC3388795

[20] Jo YH. Endogenous BDNF regulates inhibitory synaptic transmission in the ventromedial nucleus of the hypothalamus. Journal of neurophysiology. 2012;107(1):42–9. 10.1152/jn.00353.2011 21994261

[21] Barry PH, Lynch JW. Liquid junction potentials and small cell effects in patch-clamp analysis. The Journal of membrane biology. 1991;121(2):101–17. 1715403 10.1007/BF01870526

[22] Roepke TA, Qiu J, Smith AW, Ronnekleiv OK, Kelly MJ. Fasting and 17beta-estradiol differentially modulate the M-current in neuropeptide Y neurons. The Journal of neuroscience: the official journal of the Society for Neuroscience. 2011;31(33):11825–35. 10.1523/JNEUROSCI.1395-11.2011 21849543 PMC3243733

[23] Roepke TA, Smith AW, Ronnekleiv OK, Kelly MJ. Serotonin 5-HT2C receptor-mediated inhibition of the M-current in hypothalamic POMC neurons. American journal of physiology Endocrinology and metabolism. 2012;302(11):E1399–406. 10.1152/ajpendo.00565.2011 22436698 PMC3378066

[24] Korolkiewicz R, Kleinrok Z, Mlynarczyk M. Intracerebroventricular pertussis toxin enhances sensitivity to chemical convulsants and decreases the protective efficacy of carbamazepine in mice. Pharmacological research: the official journal of the Italian Pharmacological Society. 1996;33(3):211–5. 10.1006/phrs.1996.0029 8880893

[25] Funada M, Suzuki T, Narita M, Misawa M, Nagase H. Modification of morphine-induced locomotor activity by pertussis toxin: biochemical and behavioral studies in mice. Brain research. 1993;619(1–2):163–72. 8374774 10.1016/0006-8993(93)91608-u

[26] Wu ZQ, Li M, Chen J, Chi ZQ, Liu JG. Involvement of cAMP/cAMP-dependent protein kinase signaling pathway in regulation of Na+,K+-ATPase upon activation of opioid receptors by morphine. Molecular pharmacology. 2006;69(3):866–76. 10.1124/mol.105.016501 16317112

[27] Castan-Laurell I, Dray C, Attane C, Duparc T, Knauf C, Valet P. Apelin, diabetes, and obesity. Endocrine. 2011;40(1):1–9. 10.1007/s12020-011-9507-9 21725702

[28] O'Carroll AM, Lolait SJ, Harris LE, Pope GR. The apelin receptor APJ: journey from an orphan to a multifaceted regulator of homeostasis. The Journal of endocrinology. 2013;219(1):R13–35. 10.1530/JOE-13-0227 23943882

[29] Knauf C, Drougard A, Fournel A, Duparc T, Valet P. Hypothalamic actions of apelin on energy metabolism: new insight on glucose homeostasis and metabolic disorders. Hormone and metabolic research = Hormon- und Stoffwechselforschung = Hormones et metabolisme. 2013;45(13):928–34. 10.1055/s-0033-1351321 23950038

[30] Fields TA, Casey PJ. Signalling functions and biochemical properties of pertussis toxin-resistant G-proteins. The Biochemical journal. 1997;321 (Pt 3):561–71. 9032437 10.1042/bj3210561 PMC1218106

[31] Nelson TJ, Sun MK, Hongpaisan J, Alkon DL. Insulin, PKC signaling pathways and synaptic remodeling during memory storage and neuronal repair. European journal of pharmacology. 2008;585(1):76–87. 10.1016/j.ejphar.2008.01.051 18402935

[32] Katz A, Wu D, Simon MI. Subunits beta gamma of heterotrimeric G protein activate beta 2 isoform of phospholipase C. Nature. 1992;360(6405):686–9. Epub 1992/12/17. 10.1038/360686a0 1465134

[33] Clapham DE, Neer EJ. New roles for G-protein beta gamma-dimers in transmembrane signalling. Nature. 1993;365(6445):403–6. Epub 1993/09/30. 10.1038/365403a0 8413584

[34] Wu D, Katz A, Simon MI. Activation of phospholipase C beta 2 by the alpha and beta gamma subunits of trimeric GTP-binding protein. Proceedings of the National Academy of Sciences of the United States of America. 1993;90(11):5297–301. Epub 1993/06/01. 8389480 10.1073/pnas.90.11.5297 PMC46703

[35] Robbins J. KCNQ potassium channels: physiology, pathophysiology, and pharmacology. Pharmacology & therapeutics. 2001;90(1):1–19. 11448722 10.1016/s0163-7258(01)00116-4

[36] Delmas P, Brown DA. Pathways modulating neural KCNQ/M (Kv7) potassium channels. Nature reviews Neuroscience. 2005;6(11):850–62. 10.1038/nrn1785 16261179

[37] Leao RN, Tan HM, Fisahn A. Kv7/KCNQ channels control action potential phasing of pyramidal neurons during hippocampal gamma oscillations in vitro. The Journal of neuroscience: the official journal of the Society for Neuroscience. 2009;29(42):13353–64. 10.1523/JNEUROSCI.1463-09.2009 19846723 PMC6665214

[38] Parton LE, Ye CP, Coppari R, Enriori PJ, Choi B, Zhang CY, et al Glucose sensing by POMC neurons regulates glucose homeostasis and is impaired in obesity. Nature. 2007;449(7159):228–32. 10.1038/nature06098 17728716

[39] Cowley MA, Smart JL, Rubinstein M, Cerdan MG, Diano S, Horvath TL, et al Leptin activates anorexigenic POMC neurons through a neural network in the arcuate nucleus. Nature. 2001;411(6836):480–4. 10.1038/35078085 11373681

[40] Yue P, Jin H, Aillaud M, Deng AC, Azuma J, Asagami T, et al Apelin is necessary for the maintenance of insulin sensitivity. American journal of physiology Endocrinology and metabolism. 2010;298(1):E59–67. Epub 2009/10/29. 10.1152/ajpendo.00385.2009 19861585 PMC2806109

[41] Plum L, Ma X, Hampel B, Balthasar N, Coppari R, Munzberg H, et al Enhanced PIP3 signaling in POMC neurons causes KATP channel activation and leads to diet-sensitive obesity. The Journal of clinical investigation. 2006;116(7):1886–901. 10.1172/JCI27123 16794735 PMC1481658

[42] Sohn JW, Xu Y, Jones JE, Wickman K, Williams KW, Elmquist JK. Serotonin 2C receptor activates a distinct population of arcuate pro-opiomelanocortin neurons via TRPC channels. Neuron. 2011;71(3):488–97. 10.1016/j.neuron.2011.06.012 21835345 PMC3184528

[43] Sternweis PC, Smrcka AV. Regulation of phospholipase C by G proteins. Trends in biochemical sciences. 1992;17(12):502–6. 1335185 10.1016/0968-0004(92)90340-f

[44] Al-Qassab H, Smith MA, Irvine EE, Guillermet-Guibert J, Claret M, Choudhury AI, et al Dominant role of the p110beta isoform of PI3K over p110alpha in energy homeostasis regulation by POMC and AgRP neurons. Cell metabolism. 2009;10(5):343–54. 10.1016/j.cmet.2009.09.008 19883613 PMC2806524

[45] Claret M, Smith MA, Batterham RL, Selman C, Choudhury AI, Fryer LG, et al AMPK is essential for energy homeostasis regulation and glucose sensing by POMC and AgRP neurons. The Journal of clinical investigation. 2007;117(8):2325–36. 10.1172/JCI31516 17671657 PMC1934578

[46] Berglund ED, Vianna CR, Donato J Jr, Kim MH, Chuang JC, Lee CE, et al Direct leptin action on POMC neurons regulates glucose homeostasis and hepatic insulin sensitivity in mice. The Journal of clinical investigation. 2012;122(3):1000–9. 10.1172/JCI59816 22326958 PMC3287225

